# 

**Published:** 1890-05

**Authors:** 


### CONTENTS:

Article I.—Monthly meeting of the American Academy of Dental Science, held at the Boston Medical Library Associotion Rooms, February 5, 1890. - - 1

" II.—Changes of the Oral Secretions. By G. T. Thurer. 16

" III.—Some Practical points learned at Society Meetings and Clinics. By Dr. B. A. R. Ottolengui. - - 23

" IV.—A study of electricity, with the view of comprehending its application in Dentistry. By H. E. Roberts, D. D. S. - - - - - 31

### COMMUNICATIONS.

Missouri State Dental Association. - - - - - 41

Meeting of the American Dental Association. - - - 42

St. Louis Dental Society. - - - - - - - 43

### EDITORIAL.

The Dental Protective Association. - - - - - 44

Invitation to the Tenth International Medical Congress. - 46

### MONTHLY SUMMARY.

Mouth-Breathing and the Teeth. - - - - - 47

A Case in Practice. - - - - - . - 48

